# Disparities of indoor temperature in winter: A cross‐sectional analysis of the Nationwide Smart Wellness Housing Survey in Japan

**DOI:** 10.1111/ina.12708

**Published:** 2020-07-06

**Authors:** Wataru Umishio, Toshiharu Ikaga, Yoshihisa Fujino, Shintaro Ando, Tatsuhiko Kubo, Yukie Nakajima, Tanji Hoshi, Masaru Suzuki, Kazuomi Kario, Takesumi Yoshimura, Hiroshi Yoshino, Shuzo Murakami

**Affiliations:** ^1^ Department of Architecture and Building Engineering, School of Environment and Society Tokyo Institute of Technology Meguro‐ku Tokyo Japan; ^2^ Department of System Design Engineering, Faculty of Science and Technology Keio University Yokohama Kanagawa Japan; ^3^ Department of Environmental Epidemiology, Institute of Industrial Ecological Sciences University of Occupational and Environmental Health Kitakyushu Fukuoka Japan; ^4^ Department of Architecture, Faculty of Environmental Engineering University of Kitakyushu Kitakyushu Fukuoka Japan; ^5^ Department of Public Health and Health Policy, Graduate School of Biomedical and Health Sciences Hiroshima University Hiroshima Hiroshima Japan; ^6^ School of Science for Open and Environmental Systems, Graduate School of Science and Technology Keio University Yokohama Kanagawa Japan; ^7^ Japan Society for the Promotion of Science Chiyoda‐ku Tokyo Japan; ^8^ Tokyo Metropolitan University Hachioji Tokyo Japan; ^9^ Department of Emergency Medicine Tokyo Dental College Ichikawa General Hospital Ichikawa Chiba Japan; ^10^ Division of Cardiovascular Medicine, Department of Medicine Jichi Medical University School of Medicine Shimotsuke Tochigi Japan; ^11^ University of Occupational and Environmental Health Kitakyushu Fukuoka Japan; ^12^ Tohoku University Sendai Miyagi Japan; ^13^ Institute for Building Environment and Energy Conservation Chiyoda‐ku Tokyo Japan

**Keywords:** cross‐sectional analysis, health disparity, housing, indoor temperature, socioeconomic status, winter

## Abstract

The WHO Housing and health guidelines recommend a minimum indoor temperature of 18°C to prevent cold‐related diseases. In Japan, indoor temperatures appear lower than in Euro‐American countries because of low insulation standards and use of partial intermittent heating. This study investigated the actual status of indoor temperatures in Japan and the common characteristics of residents who live in cold homes. We conducted a nationwide real‐world survey on indoor temperature for 2 weeks in winter. Cross‐sectional analyses involving 2190 houses showed that average living room, changing room, and bedroom temperatures were 16.8°C, 13.0°C, and 12.8°C, respectively. Comparison of average living room temperature between prefectures revealed a maximum difference of 6.7°C (Hokkaido: 19.8°C, Kagawa: 13.1°C). Compared to the high‐income group, the odds ratio for living room temperature falling below 18°C was 1.38 (95% CI: 1.04‐1.84) and 2.07 (95% CI: 1.28‐3.33) for the middle‐ and low‐income groups. The odds ratio was 1.96 (95% CI: 1.19‐3.22) for single‐person households, compared to households living with housemates. Furthermore, lower room temperature was correlated with local heating device use and a larger amount of clothes. These results will be useful in the development of prevention strategies for residents who live in cold homes.

**Practical implications**

- In modern society, humans spend 60%‐70% of their time at home; housing thermal environment is of great importance to health.
- We actually measured indoor temperature of 2190 houses throughout Japan for 2 weeks in winter.
- There were disparities in living room temperature within Japan, and they related to socioeconomic status, single‐person households and clothing and daily lifestyle habits.
- These housing disparities have the potential to cause health disparities due to cold‐related diseases.
- We expect these findings will be useful in the reduction in health disparities.

## 1 BACKGROUND

In recent years, clear health disparities have been identified not only between nations, but also within nations. These cannot be ignored, and reducing them has become a global issue. The World Health Organization (WHO), which promotes the concept of “Health for all,” emphasizes the importance of the social determinants of health^1^—namely the conditions in which people are born, raised, work, live, and age—to correct these disparities.^2^ Even in Japan, the National Health Promotion Movement in the 21st Century (Health Japan 21 (the Second Term)) holds “reduction of health disparities” and “establishment of a social environment” as its key values and states that the “health of an individual is affected by such social environment as family, schools, the community, and workplaces.^3^” In particular, given that humans spend 60%‐70% of their time at home,^4^, ^5^, ^6^ and that elderly people with declining physiological function or children with undeveloped physiological function spend even more time at home,^7^ the living environment at home is of great importance to health.

The WHO issued Housing and health guidelines in October 2018.^8^ One of the five priority areas of the guideline is “low indoor temperatures and insulation.” The guideline shows evidence that cold indoor temperatures have adverse consequences for health; as an example, the number of excess winter deaths due to cold housing has been estimated at 38 200 per year (12.8/100 000) in 11 selected European countries.^9^ The guideline systematically reviewed evidence and recommends a minimum indoor temperature of 18°C, stating that “there is high certainty that taking measures to warm cold houses will have significant health benefits and a minimum of 18°C is widely accepted.” While an investigation in the UK^10^, ^11^ and USA^12^ showed that the average living room temperature during winter exceed 18°C, an investigation in Japan^13^ reported that the average living room temperature during winter was 17°C, indicating that many houses did not achieve the recommended indoor temperature. Accordingly, there is concern that indoor temperature may greatly affect health, especially in Japan.

Why are indoor temperatures in Japanese houses lower than those in European and American houses? The difference appears to be due to two factors—low insulation levels and differences in heating use. According to an estimation by the Ministry of Land, Infrastructure, Transport and Tourism, 39% of about 50 million existing houses in Japan are not insulated and only 5% of those are relatively well insulated to meet the 1999 standards (the highest thermal insulation standards in Japan).^14^ In addition, while continuous heating of the entire building is the norm in Europe and the USA, partial intermittent heating in the living room only is the norm in Japan. As a result, energy consumption in houses in Japan is minimal compared to that of other countries, with energy used for heating only one‐quarter of that used in European and American countries.^15^

Based on these background factors, we conducted a nationwide survey named the “Smart Wellness Housing (SWH) survey” in Japan which aimed to quantitatively evaluate the relationship between housing and health. A previous report^16^ from this survey revealed that systolic blood pressure in the morning and evening increased by 8.2 mmHg and 6.5 mmHg per 10°C decrease in indoor temperature, indicating that the risk of hypertension increased at low indoor temperature. Therefore, it is considered useful from a population strategy viewpoint to discover “who lives in a cold home (what are the common characteristics of residents who live in low indoor temperature environments) in real‐world settings.” The present results were therefore focused on the relationship between the characteristics of residents and indoor temperature.

## 2 METHODS

### 2.1 Climate area classification in Japan

**FIGURE 1:**
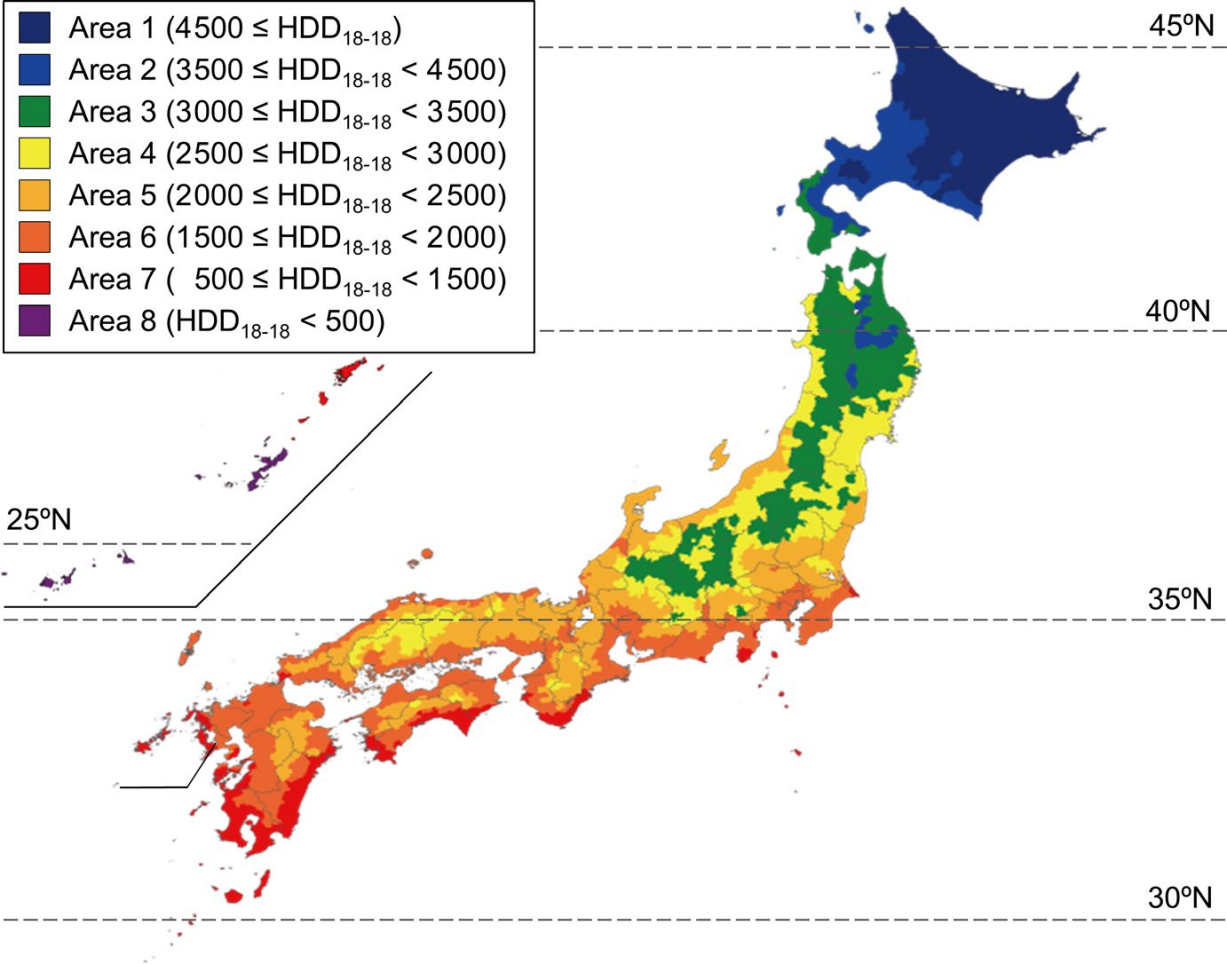
Eight climate areas in Japan

In Japan, a total of eight climate areas are defined under the law based on the heating degree‐days (HDD_18‐18_) value (difference between indoor temperature of 18°C and daily mean outdoor temperature, where the daily mean outdoor temperature is less than 18°C; Figure S1). The eight climate areas are shown in Figure 1. The required insulation standards have been defined for each climate area in Japan. The analysis in this paper examined this climate area classification under the variable “climate area.” An approximate climate area classification at the prefectural level is shown in Table 1. A detailed climate area classification at the municipal level has been reported elsewhere.^17^

**TABLE 1:** Climate area classification at the prefectural level based on heating degree‐days value

Area	HDD_18‐18_ ^a^ [°C day]	Prefecture in climate area classification
1	4500 ≤ HDD_18‐18_	Hokkaido
2	3500 ≤ HDD_18‐18_ < 4500	
3	3000 ≤ HDD_18‐18_ < 3500	Aomori, Iwate, Akita
4	2500 ≤ HDD_18‐18_ < 3000	Miyagi, Yamagata, Fukushima, Tochigi, Niigata, Nagano
5	2000 ≤ HDD_18‐18_ < 2500	Ibaraki, Gunma, Saitama, Chiba, Tokyo, Kanagawa, Toyama, Ishikawa, Fukui, Yamanashi, Gifu, Shizuoka, Aichi, Mie, Shiga, Kyoto, Osaka, Hyogo, Nara, Wakayama, Tottori, Shimane, Okayama, Hiroshima, Yamaguchi, Tokushima, Kagawa, Ehime, Kochi, Fukuoka, Saga, Nagasaki, Kumamoto, Oita
6	1500 ≤ HDD_18‐18_ < 2000	
7	500 ≤ HDD_18‐18_ < 1500	Miyazaki, Kagoshima
8	HDD_18‐18_ < 500	Okinawa

^a^ HDD_18‐18_ indicates heating degree‐days.

### 2.2 Study design

The SWH survey was administered to households that had the intention of conducting insulation retrofitting of their homes. This survey was conducted as a non‐randomized controlled trial with groups defined according to participants’ choice to actually conduct or not conduct insulation retrofitting. The study protocol and informed consent procedure were approved by the ethics committee of the Hattori Clinic Institutional Review Board. All of the participants provided written informed consent to participate and to have their data published.

Since we focused mainly on the health effects of insulation retrofitting in the SWH survey, inclusion criteria were (a) intention to conduct insulation retrofitting, (b) age over 20 years, and (c) pre‐retrofitting house did not meet S (Supreme) standards of the “Act on the Promotion of Dissemination of Long‐Lasting Quality Housing” in Japan.^18^ A summary of the S standards is shown in Tables S2‐S4. For example, a house with an average heat transmission coefficient for the outer skin (U_A_ value) of 0.87 W/m^2^K or less in Tokyo meets the S standards. A standard value of heat transmission coefficient for windows (U_W_ value) of 4.65 W/m^2^K or less in Tokyo is required to meet the S standards. For reference, the prescriptive requirements of U_W_ value in California, which is located at about the same latitude as Tokyo, is 1.8 W/m^2^K,^19^ while that of a notional dwelling in the UK is 1.4 W/m^2^K.^20^

Participants were recruited through construction companies throughout all 47 prefectures of Japan (Figure S2). After the recruitment of households, the Japan Sustainable Building Consortium, the main body governing the research project, submitted a request to conduct the investigation. Households that gave consent were sent an investigation kit that included questionnaires, a thermo‐hygrometer and an HBP meter, with written instructions on how to use them. The participants started the 14‐day survey within 5 days of receiving the investigation kit. Two participants per household (generally a husband and wife pair) were asked to conduct actual measurements.

In this paper, we performed a cross‐sectional analysis of data from the baseline (before insulation retrofitting) survey in the winter seasons (November‐March) of 2014 to 2019. We focused on data before insulation retrofitting to reflect the actual condition of houses in Japan, most of which have a low insulation level.^14^

### 2.3 Indoor temperature and other measurements

Indoor temperature and relative humidity at 1.0 m above the floor were measured in the living room, changing room (the room the resident typically used to changed clothes), and bedroom at 10‐min intervals for 2 weeks (TR‐72wf; T&D Corp.). The logger has the following specification: ranges of temperature and relative humidity were 0‐55°C and 10%‐95%RH, accuracy of ±0.5°C and ±5%RH, and resolution of 0.1°C and 1%RH. We chose the logger in consideration of indoor temperature and humidity distributions in the previous survey in Japan,^13^ which showed almost all data were within 0‐30°C and 10%‐90%RH. There were two cautions regarding installation site of the logger, namely that (a) it was not placed in direct sunlight and (b) was far away from heating equipment or heat‐generating devices like refrigerators and televisions. Outdoor temperature (Temp_Out_) was obtained from the closest local meteorological observatory to each participant's house.

Participants were also asked to measure their home blood pressure (HBP) in the living room, twice after getting out of bed in the morning and twice before getting into bed in the evening (HEM‐7251G; Omron Healthcare Co., Ltd.). The clock time of the HBP measurement was automatically stored with the HBP data and uploaded to the internet via 3G mobile networks. We used the living room temperature at the clock time of HBP measurement in the morning in the analysis, on the basis that cardiovascular events frequently occur in the morning.^21^, ^22^

A questionnaire survey was conducted and enquired about individual attributes, such as age, sex, and weight; socioeconomic status, such as household income, single‐person household; and lifestyle, such as kotatsu use (traditional Japanese local heating device, Figure S3) and patterns of clothes worn. Household income was chosen from multiple choices that defined the range of household income (<0.5 million JPY, 0.5‐1 million JPY, …, ≥10 million JPY) and classified as low (<2 million JPY), middle (2‐6 million JPY), and high (≥6 million JPY) in reference to the National Health and Nutrition Survey, led by the Ministry of Health, Labour and Welfare. Patterns of clothes were chosen from multiple‐choice selections, and the amount of clothes was calculated in accordance with ISO 7730:2005^23^ and ANSI/ASHRAE Standard 55‐2013^24^ (Figure S4). A diary survey was also conducted, in which participants provided details of their waking time, bedtime, and time spent at home on a daily basis. These data were used for extraction of indoor temperature at home and during sleep in the following analysis.

### 2.4 Statistical analysis

The relationship between characteristics of heads of households and indoor temperature was analyzed by multilevel linear regression. The dependent variable was living room temperature at the time of HBP measurement in the morning. The model was developed with random intercepts, consisting of two levels: repeatedly measured day‐level variables (Temp_Out_) were nested within household‐level variables (age, duration of residence in the house, household income, single‐person households, kotatsu use, amount of clothes, and climate area). These variables were selected based on a univariate analysis of average living room temperature which showed differences between groups at a two‐sided *P* value < 0.05 (Table S5). The variable “sex” was strongly correlated with single‐person households (*r* = 0.41) and was therefore excluded in consideration of multicollinearity. Multilevel logistic regression analysis was also performed (dependent variable: living room temperature in the morning < 18°C or not). Details of the model are shown in the online supplementary file (Appendix S2). Day‐level variables were centered around means for individual households, while household‐level variables were centered around the overall mean. Regression coefficients were estimated using the maximum likelihood method. All *P* values were two sided, and a two‐sided *P* value less than 0.05 was considered statistically significant. All analyses were performed using SPSS Ver. 25 (SPSS Inc, Chicago, IL, USA).

## 3 RESULTS

### 3.1 Baseline characteristics of residents and indoor temperatures

Table 2 shows the characteristics of heads of households in 2190 houses. The average age was 59 years, and 81.5% were men. The average duration of residence in a house is 27 years. A national census in Japan noted that the most frequent duration of residence was 20 years or more, so it is considered that this result reflects actual conditions in Japan. The average amount of clothes is 0.95 clo, which is approximately equivalent to underpants, shirt, trousers, jacket, socks, and shoes (1.00 clo).^23^ About one‐third of households had high household income (≥6 million JPY); about 10% of households were single‐person households; and about 40% of households used kotatsu. Area 6 is the most populated area among the eight climate areas and was used as a reference variable in the following analysis.

**TABLE 2:** Characteristics of heads of households in the baseline survey

Variable	Mean	SD
Age, years	59.4	13.3
Body mass index, kg/m^2^	23.5	3.6
Duration of residence in house, years	26.8	17.0
Amount of clothes, clo^a^	0.95	0.20

Variable	Number	%
Sex		
Men	1784	81.5
Women	406	18.5
Household income		
Low (<2 million JPY)	259	12.8
Middle (2‐6 million JPY)	1041	51.3
High (≥6 million JPY)	730	36.0
Number of housemates		
1 (single‐person household)	213	9.9
≥2	1931	90.1
Kotatsu^b^ use		
None	1304	60.2
Currently used	862	39.8
Climate area		
Area 2	69	3.2
Area 3	69	3.2
Area 4	250	11.4
Area 5	562	25.7
Area 6	1109	50.7
Area 7	130	5.9

^a^ clo is a unit that represents the thermal resistance of clothes. 1 clo = 0.155(m^2^K)/W.

^b^ Traditional Japanese local heating device, consisting of a low table with an electric heater attached to the underneath surface and covered by a thick blanket.

2, 3, 4 show the average and minimum temperatures in the living room, changing room and bedroom when participants were at home or during sleep (maximum temperature is shown in Figure S5). The average temperature in the living room and changing room was 16.8°C and 13.0°C when participants were at home (excluding the period during sleep), and the average temperature in the bedroom was 12.8°C during sleep. The minimum temperature in the living room, changing room, and bedroom was 12.6°C, 10.4°C, and 11.2°C on average, and was below 18°C (the recommended minimum temperature by the WHO guidelines) in more than 90% of households.

**FIGURE 2:**
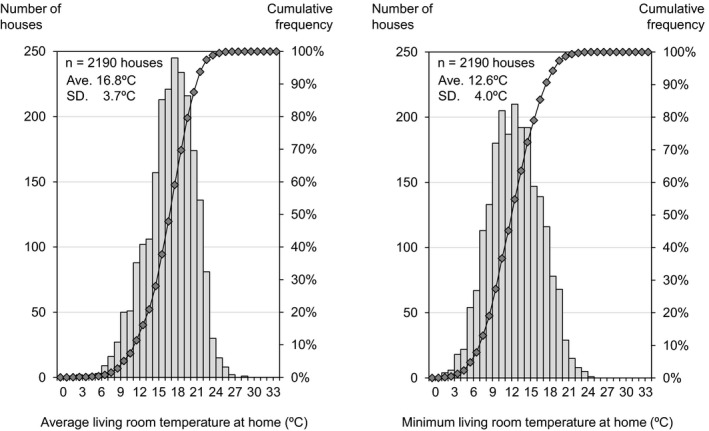
Average (left) and minimum (right) living room temperature at home. ^†^The bar chart indicates the number of houses and the line chart indicates cumulative frequency. The result shows the 2‐wk average of daily average/minimum temperature for each house when participants were at home (excluding the period during sleep)

**FIGURE 3:**
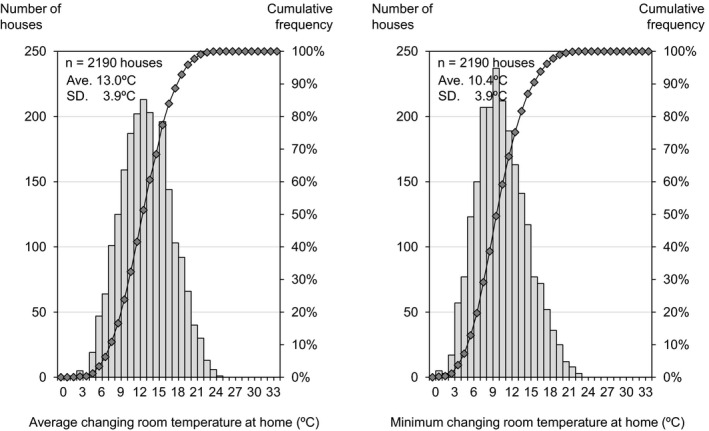
Average (left) and minimum (right) changing room temperature at home. ^†^The bar chart indicates the number of houses and the line chart indicates cumulative frequency. The result shows the 2‐wk average of daily average/minimum temperature for each house when participants were at home (excluding the period during sleep)

**FIGURE 4:**
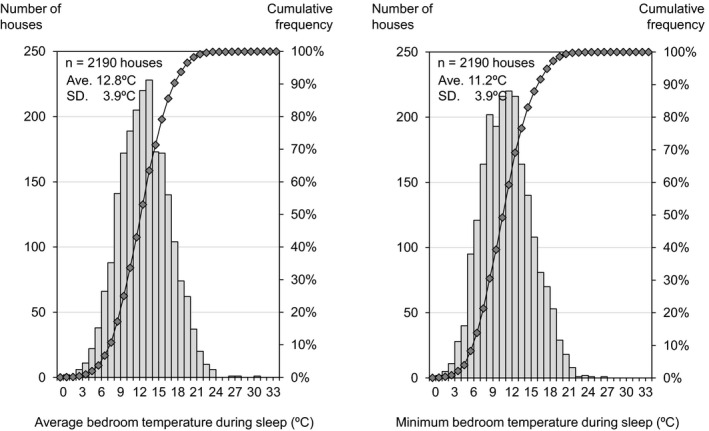
Average (left) and minimum (right) bedroom temperature during sleep. ^†^The bar chart indicates the number of houses and the line chart indicates cumulative frequency. The result shows the 2‐wk average of daily average/minimum temperature for each house when participants were in bed

**FIGURE 5:**
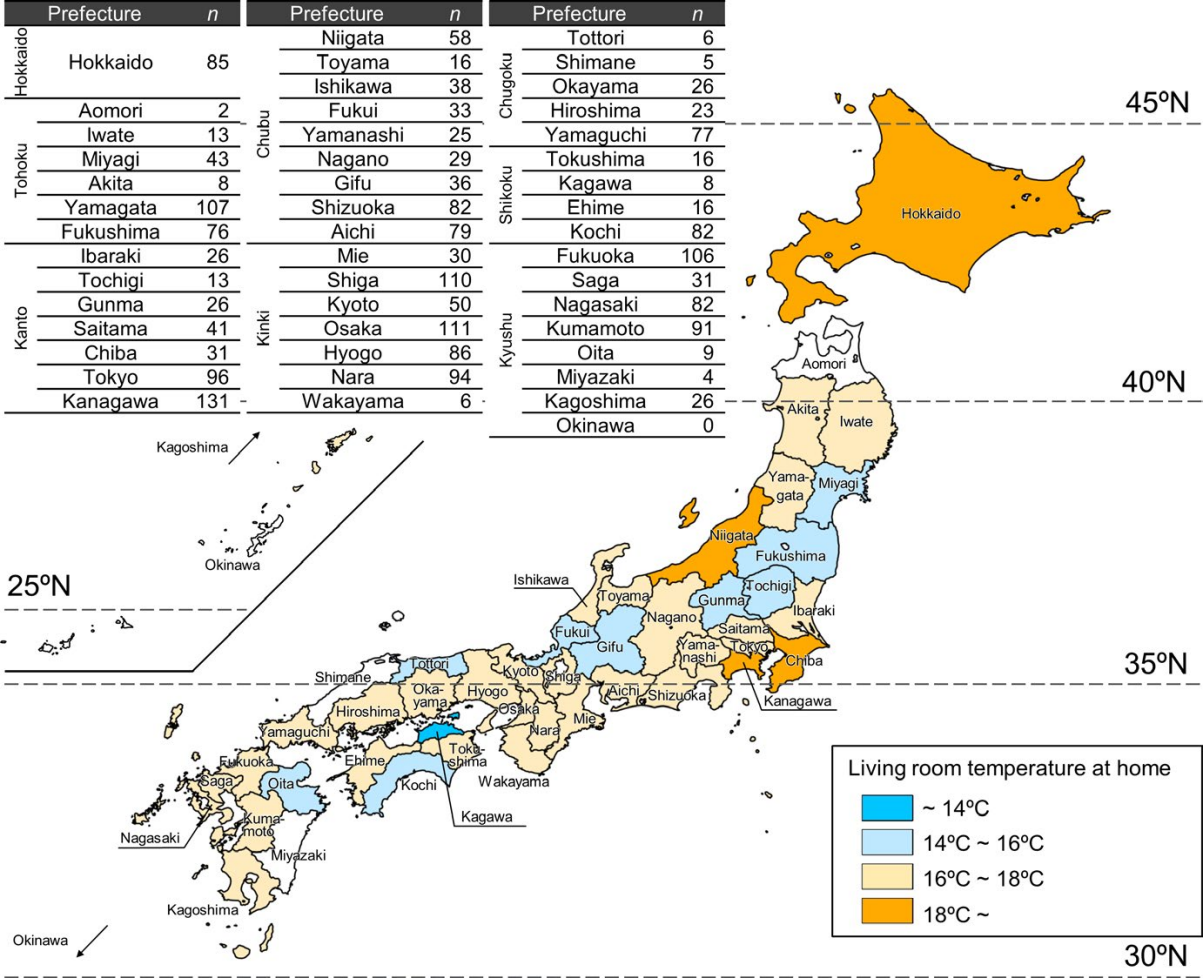
Average living room temperature at home in each prefecture. ^†^Excluding prefectures with 5 households or less (displayed in white). The result shows the 2‐wk average of daily average temperature for each house when participants were at home (excluding the period during sleep)

The average living room temperature in each prefecture is shown in Figure 5, excluding prefectures with 5 households or less. Of 43 prefectures, temperatures exceeded 18°C in only 4 prefectures (Hokkaido, Chiba, Kanagawa, and Niigata). Average living room temperature was highest (19.8°C) in Hokkaido, where outdoor temperature is lower and houses have a higher thermal insulation level than other areas. In contrast, average living room temperature was lowest (13.1°C) in Kagawa, which is located in the southwestern area of Japan and considered to have a mild climate.

### 3.2 Identification of residents living in low indoor temperature environments

The results of multilevel linear regression analyses are shown in Table 3. Regarding day‐level variable, outdoor temperature was positively correlated with living room temperature (*P* < .001). A 1°C decrease in outdoor temperature was associated with a 0.33°C decrease in living room temperature in the morning. Regarding household‐level variables, living room temperature in climate area 2 was significantly higher by 3.7°C compared with area 6. In contrast, indoor temperature was significantly lower by 1.2°C in area 4 compared with area 6. Lower household income was significantly correlated with lower living room temperatures. Compared to the high income (≥6 million JPY) group, living room temperature in the morning was 0.5°C lower for the middle income (2‐6 million JPY) group and 1.1°C lower for the low income (<2 million JPY) group. Single‐person households lived in a 1.4°C lower environment than households living with housemates. Kotatsu use was associated with 1.5°C lower living room temperature than non‐kotatsu use. Moreover, longer period of residence in the same house (0.4°C decrease/10 year increase) and wearing larger amounts of clothes (0.3°C decrease/0.1clo increase) were correlated with lower living room temperatures.

**TABLE 3:** Multilevel linear regression model of living room temperature in the morning

Explanatory variable	Univariate model			Multivariate model		
	*β*	(95%CI)	*P* value	Adjusted *β*	(95%CI)	*P* value
Level 1: Day‐level variable						
Temp_Out_ [°C]	0.33	(0.33, 0.34)	<0.001	0.33	(0.33, 0.34)	<0.001
Level 2: Household‐level variable						
Age [years]	−0.05	(−0.06, −0.03)	<0.001	0.01	(−0.01, 0.03)	0.336
Duration of residence [years]	−0.06	(−0.07, −0.05)	<0.001	−0.04	(−0.05, −0.03)	<0.001
Household income						
High (≥6 million JPY)	ref.	－	－	ref.	－	－
Middle (2‐6 million JPY)	−0.78	(−1.22, −0.33)	<0.001	−0.51	(−0.94, −0.09)	0.018
Low (<2 million JPY)	−1.75	(−2.44, −1.07)	<0.001	−1.10	(−1.78, −0.41)	0.002
Number of housemates						
≥2	ref.	－	－	ref.	－	－
1 (single − person)	−1.38	(−2.12, −0.64)	<0.001	−1.37	(−2.08, −0.67)	<0.001
Amount of clothes [clo]^a^	−4.06	(−5.10, −3.02)	<0.001	−2.85	(−3.87, −1.83)	<0.001
Kotatsu^b^ use						
None	ref.	－	－	ref.	－	－
Currently used	−2.13	(−2.54, −1.71)	<0.001	−1.50	(−1.91, −1.10)	<0.001
Climate area						
Area 6	ref.	－	－	ref.	－	－
Area 2	4.01	(2.80, 5.22)	<0.001	3.66	(2.50, 4.83)	<0.001
Area 3	−1.05	(−2.28, 0.18)	0.094	−0.59	(−1.76, 0.58)	0.323
Area 4	−1.80	(−2.46, −1.15)	<0.001	−1.21	(−1.84, −0.58)	<0.001
Area 5	−0.81	(−1.30, −0.32)	0.001	−0.62	(−1.09, −0.16)	0.009
Area 7	−0.31	(−1.18, 0.55)	0.478	−0.04	(−0.86, 0.78)	0.926
Intercept	15.03	(15.11, 15.68)	<0.001	16.36	(15.97, 16.76)	<0.001

Note

n = 22 518 observations (1701 households × ave. 13 observations/household) CI indicates confidence interval; Temp_Out_, outdoor temperature.

^a^ clo is a unit that represents the thermal resistance of clothes. 1 clo = 0.155(m^2^K)/W.

^b^ Traditional Japanese local heating device, consisting of a low table with an electric heater attached to the underneath surface and covered by a thick blanket.

The results of multilevel logistic regression analyses are shown in Table 4. Compared to the high income (≥6 million JPY) group, the odds ratio for living room temperature falling below 18°C was 1.38 (95% CI: 1.04‐1.84) for the middle income (2‐6 million JPY) group and 2.07 (95% CI: 1.28‐3.33) for the low income (<2 million JPY) group. The odds ratio was 1.96 (95% CI: 1.19‐3.22) for single‐person households compared to households living with housemates. Kotatsu use (odds ratio = 2.60; *P* < .001) and wearing larger amounts of clothes (odds ratio = 6.86; *P* < .001) were also correlated with lower living room temperatures.

**TABLE 4:** Odds ratio for living room temperature in the morning falling below 18˚C

Explanatory variable	Univariate model			Multivariate model		
	Odds ratio	(95%CI)	*P* value	Odds ratio	(95%CI)	*P* value
Level 1: Day‐level variable						
Temp_Out_ [°C]	0.83	(0.82, 0.84)	<0.001	0.82	(0.81, 0.83)	<0.001
Level 2: Household‐level variable						
Age [years]	1.02	(1.02, 1.03)	<0.001	1.00	(0.98, 1.01)	0.622
Duration of residence [years]	1.03	(1.03, 1.04)	<0.001	1.02	(1.01, 1.03)	<0.001
Household income						
High (≥6 million JPY)	ref.	－	－	ref.	－	－
Middle (2‐6 million JPY)	1.38	(1.07, 1.77)	0.014	1.38	(1.04, 1.84)	0.026
Low (<2 million JPY)	2.36	(1.60, 3.49)	<0.001	2.07	(1.28, 3.33)	0.003
Number of housemates						
≥2	ref.	－	－	ref.	－	－
1 (single‐person)	1.76	(1.17, 2.64)	0.006	1.96	(1.19, 3.22)	0.008
Amount of clothes [clo]^a^	10.41	(5.95, 18.22)	<0.001	6.86	(3.45, 13.64)	<0.001
Kotatsu^b^ use						
None	ref.	－	－	ref.	－	－
Currently used	3.51	(2.78, 4.43)	<0.001	2.60	(1.98, 3.43)	<0.001
Climate area						
Area 6	ref.	－	－	ref.	－	－
Area 2	0.08	(0.04, 0.15)	<0.001	0.08	(0.04, 0.17)	<0.001
Area 3	1.34	(0.68, 2.63)	0.398	1.35	(0.60, 3.05)	0.470
Area 4	1.73	(1.19, 2.52)	0.004	1.17	(0.76, 1.81)	0.467
Area 5	1.47	(1.12, 1.93)	0.005	1.33	(0.97, 1.83)	0.077
Area 7	1.38	(0.86, 2.24)	0.184	1.16	(0.67, 2.02)	0.595
Intercept	3.90	(3.48, 4.37)	<0.001	2.24	(1.71, 2.92)	<0.001

Note

n = 22 518 observations (1701 households × ave. 13 observations/household) CI indicates confidence interval; Temp_Out_, outdoor temperature.

^a^ clo is a unit that represents the thermal resistance of clothes. 1 clo = 0.155(m^2^K)/W.

^b^ Traditional Japanese local heating device, consisting of a low table with an electric heater attached to the underneath surface and covered by a thick blanket.

## 4 DISCUSSION

### 4.1 Summary of findings

This study analyzed the relationship between living room temperature in the winter season and the characteristics of residents based on a baseline (before insulation retrofitting) survey of 2190 households. The cross‐sectional analysis showed that (a) the average temperature in the living room and changing room was 16.8°C and 13.0°C when participants were at home (excluding the period during sleep), and the average temperature in the bedroom was 12.8°C during sleep; (b) the average living room temperature was highest (19.8°C) in Hokkaido, where outdoor temperature is lower than other areas, but lowest (13.1°C) in Kagawa, which is considered to have a mild climate; (c) lower household income and single‐person households were correlated with lower indoor temperatures; and (d) use of a kotatsu (traditional Japanese local heating device) and wearing larger amounts of clothes were correlated with lower indoor temperatures.

### 4.2 Housing disparities between nations and within Japan

The results of field measurements of indoor temperature from 2190 houses across Japan indicate that the average living room temperature in winter was 16.8°C. Similarly, as described in the background, the average living room temperature of 602 houses across Japan was 17°C during winter.^13^ In contrast, a review of measured indoor temperature in UK homes reported that the average living room temperature in winter was 18‐21°C.^10^ In addition, the Energy Follow‐Up Survey 2011 involving 823 dwellings in the UK revealed that the mean monthly temperature for the whole house/apartment was 19.3°C during the heating season (October to April).^11^ Furthermore, an investigation that targeted apartments in New York reported an average living room temperature in winter of 23.3°C.^12^ In the bedroom, the difference in indoor temperature was more remarkable (12.8°C in the present survey in Japan and 18.9°C in the survey in the UK.^11^) Therefore, there are clear housing disparities between nations in winter. This is because Japanese have historically focused on a hot and humid summer when they build houses. A major medieval Japanese essay includes the description that “a house should be built with the summer in mind. In winter it is possible to live anywhere, but a badly made house is unbearable when it gets hot.” A second factor in housing disparity between Japan and other countries is the stereotypical idea among Japanese that the value of a house declines year after year from the time of construction. Construction practices and inputs are based around this perception, resulting in a society‐wide focus on low‐performance, low‐cost houses. A survey conducted by the Ministry of Land, Infrastructure, Transport and Tourism revealed that the value of a wooden house is essentially zero at 20 years after construction in Japan.^25^

The present analysis also showed that there is a major disparity in indoor temperatures even within Japan. Comparison of indoor temperature between prefectures revealed a maximum difference of 6.7°C (average living room temperature in Hokkaido minus that in Kagawa). In addition, the results of multilevel analysis showed that the living room temperature in climate area 2 (which mostly consists of Hokkaido) was significantly higher—by 3.7°C—compared with that in area 6 (which accounts for the majority of Japan, including Tokyo) after adjusting potential confounders. Even in Japan, continuous heating of the entire building is uniquely widespread in Hokkaido, because the comparatively high thermal insulation level in Hokkaido provides high heating efficiency. Thus, a regional difference in attitude toward heating was a contributory factor to these housing disparities within Japan.

WHO has emphasized the risk of low indoor temperature to health in its guideline.^8^ Consistent with this, concern has been raised that housing disparities between and within nations have the potential to cause health disparities.^26^, ^27^ In the UK, a guideline on the recommended minimum indoor temperature of 18°C was issued,^28^ while in the northeastern states of the USA, the minimum room temperature is regulated by law (eg, between 6 AM and 10 PM, at least 20.0°C (68°F), between 10 PM and 6 AM, at least 16.7°C (62°F) in New York State).^29^ Referring to these examples of best practice from other countries, there may still be scope for an improvement in insulation standards in Japan, and there is a need to establish indoor temperature standards. We expect that our present results about disparities of indoor temperature will contribute to regulatory developments in Japan and a reduction in health disparities.

### 4.3 Residents at high risk of hypertension and cardiovascular disease

Our previous analyses^16^ indicated that indoor temperature is strongly related to blood pressure and that hypertension occurs in low room temperature environments. Furthermore, hypertension during the winter season can lead to the development of cardiovascular diseases (CVDs) and has been known to cause excess winter deaths.^30^, ^31^ In other words, residents living in low room temperature environments have a high risk of hypertension and CVDs.

Studies of the determinants of room temperature conducted in the UK^32^, ^33^ suggest that resident attributes such as house composition and employment status significantly affect room temperature. Our present analysis showed that low household income, single‐person households, and lifestyle factors such as the use of heating and amount of clothes worn were also associated with the indoor temperature. Low household income may force residents to limit the use of heating or to live in housing with low insulation level. Recent research has shown that fuel‐poverty households exist in Japan^34^: A certain number of fuel‐poverty households are unable to afford heating expenses and accordingly live in cold homes.^35^ Our data also confirmed that the indoor temperature is lower in single‐person households. A previous study^36^ suggested that people living alone have a higher risk of hypertension, and low room temperature is hypothesized to be a contributing factor. Households using kotatsu had lower indoor temperatures. Kotatsu is a form of local heating which does not allow heating of an entire room or house. Similarly, wearing more clothes was correlated with lower indoor temperatures, which suggests that some residents attempt to brave the cold by using kotatsu or wearing more clothes.

The method used in this study allowed the identification of residents who live in cold homes after adjusting for outdoor temperature and climate areas. We expect that these results will be useful in the development of prevention strategies for these residents.

### 4.4 Strengths and limitations

The strengths of this study are following three points. First, this is one of the largest surveys on the relationship between the characteristics of residents and indoor temperature in winter, involving 2190 households in Japan. Second, participants were recruited throughout all 47 prefectures of Japan. Since the results were not obtained from a sample in a specific area, the risk of bias appears low, and the results are likely highly generalizable. Third, field measurements on indoor temperature have been conducted for 2 weeks in real‐world settings. This objective data for a certain period of time may reduce observational bias.

This study has the following limitations. First, the sample in the SWH survey was skewed toward households which had a relatively low thermal insulation level. However, in Japan, the majority of existing houses have low insulation level,^14^ so the present results may represent well the “big picture” of actual conditions in Japan. Second, we determined a target sample size based on “the effect of insulation retrofitting on HBP,” which is main theme of the SWH survey, so the sample size was small in some climate areas. This might explain why the difference due to climate area classification was not observed in some areas in the multilevel model. Thus, more precise detection of housing disparities within Japan will require an increase in sample size in these areas. Third, we were unable to survey details of the design specifications of participants’ houses because few residents had retained design drawings of their house, and they were generally unfamiliar with the installed insulation. We took the age of the house into consideration by adding the duration of residence into the multilevel model as an independent variable. However, it is necessary to consider floor area, house structure, and so on for more detailed analysis. We therefore suggest that future research should be done in cooperation with housing experts. Fourth, we focused on the relationship between characteristics of residents and indoor temperature in winter in the present analysis because excess cold causes cardiovascular and respiratory diseases. Given that excess heat in summer causes heatstroke inside the house,^37^ however, it is also necessary to conduct analyses on indoor temperature throughout the year. Finally, although indoor air quality is closely associated with health,^38^, ^39^ we could not measure household air pollutants in this survey. Further studies on total indoor environment factors such as indoor temperature, humidity, and air pollutants are needed to clarify ideal housing environment.

## 5 CONCLUSIONS

This real‐world survey involving 2190 households in Japan showed that the minimum indoor temperature in winter was below 18°C (the recommended minimum temperature by the WHO guidelines) in more than 90% of households. Also, there were disparities in living room temperature within Japan, and they related to socioeconomic status, single‐person households, and the way of living. These housing disparities have the potential to cause health disparities. We expect these results will be useful in the development of prevention strategies for residents who live in cold homes and the reduction in health disparities.

## CONFLICT OF INTEREST

TI has received research grants from Tokyo Gas Co., Ltd., Osaka Gas Co., Ltd., HyAS & Co. Inc, Fuyo Home Co. Ltd., Asahi Kasei Homes Corp., OM Solar Co. Inc, Kajima Corp., Shimizu Corp., Nice Corp., Japan Gas Association, and Japan Sustainable Building Consortium. TH has received an honorarium from LIXIL Corp. MS has received non‐restrictive research funds from Taiyo Nippon Sanso Corp. KK received a research grant from Omron Healthcare Co., Ltd. The other authors have no competing interests.

## AUTHOR CONTRIBUTIONS

**Wataru Umishio**: Conceptualization‐Equal, Data curation‐Lead, Formal analysis‐Lead, Methodology‐Equal, Validation‐Equal, Visualization‐Lead, Writing‐original draft‐Lead. **Toshiharu Ikaga**: Conceptualization‐Equal, Funding acquisition‐Equal, Methodology‐Equal, Project administration‐Equal, Supervision‐Supporting, Validation‐Equal, Writing‐review & editing‐Supporting. **Yoshihisa Fujino**: Conceptualization‐Equal, Methodology‐Equal, Validation‐Equal, Writing‐review & editing‐Lead. **Shintaro Ando**: Conceptualization‐Equal, Methodology‐Equal, Validation‐Supporting, Writing‐review & editing‐Supporting. **Tatsuhiko Kubo**: Conceptualization‐Supporting, Methodology‐Equal, Writing‐review & editing‐Supporting. **Yukie Nakajima**: Formal analysis‐Supporting, Visualization‐Supporting, Writing‐review & editing‐Supporting. **Tanji Hoshi**: Methodology‐Supporting, Writing‐review & editing‐Supporting. **Masaru Suzuki**: Validation‐Supporting, Writing‐review & editing‐Supporting. **Kazuomi Kario**: Supervision‐Supporting, Writing‐review & editing‐Supporting. **Takesumi Yoshimura**: Supervision‐Supporting, Writing‐review & editing‐Supporting. **Hiroshi Yoshino**: Supervision‐Supporting, Writing‐review & editing‐Supporting. **Shuzo Murakami**: Conceptualization‐Equal, Funding acquisition‐Lead, Project administration‐Equal, Supervision‐Lead, Writing‐review & editing‐Supporting.

### Peer Review

The peer review history for this article is available at https://publons.com/publon/10.1111/ina.12708.

## Supporting information

Supplementary Material

Supplementary Material
