# Supplementary material for: Disparities of indoor temperature in winter: A cross‐sectional analysis of the Nationwide Smart Wellness Housing Survey in Japan
Source: Indoor Air. 2020 Jul 6;30(6):1317–28. doi: 10.1111/ina.12708 (PMC7689703; doi:10.1111/ina.12708)
Supplement: Supplementary file 1 — Supplementary Material [file INA-30-1317-s001.docx]

**Supplementary Material**

**Supplement to:**

**Disparities of Indoor Temperature in Winter: A Cross-Sectional Analysis of the Nationwide Smart Wellness Housing Survey in Japan**

Wataru Umishio, Toshiharu Ikaga, Yoshihisa Fujino, Shintaro Ando, Tatsuhiko Kubo, Yukie Nakajima, Tanji Hoshi, Masaru Suzuki, Kazuomi Kario, Takesumi Yoshimura, Hiroshi Yoshino, Shuzo Murakami; on behalf of the Smart Wellness Housing survey group

**Appendix S1: Supplement tables and figures**

Table S1. Members of Smart Wellness Housing Survey Group

Table S2. Summary of S standards of the “Act on the Promotion of Dissemination of Long-Lasting Quality Housing” in Japan

Table S3. Performance-based grades of energy conservation

Table S4. Performance-based grades of thermal insulation

Table S5. Average living room temperature in the morning by resident characteristic

Figure S1. Definition of heating degree days

Figure S2. Prefectures in Japan

Figure S3. Traditional Japanese heating device “kotatsu table”

Figure S4. Patterns of clothes in the questionnaire

Figure S5. Maximum living room and changing room temperature at home

and Maximum bedroom temperature during sleep

**Appendix S2: Details of the multilevel model**

Table S1. Members of Smart Wellness Housing Survey Group

A: Members of the Research Committee for the Promotion of Smart Wellness Housing

| **Chairperson**  　Shuzo MURAKAMI *  **Vice-chairperson**  　Takesumi YOSHIMURA *  Kazuomi KARIO *  Hiroshi YOSHINO *  **Organizer**  　Toshiharu IKAGA *  **Committee member in medicine**  　Suminori AKIBA, Mikio ARITA, Michiya IGASE, Masayoshi ICHIBA, Nami IMAI, Masaki UEMURA, Hiroyuki UEHARA, Haruo UGUISU, Kensuke ESATO, Akira EBOSHIDA, Kuniaki OTSUKA *, Yuko OGUMA, Toshiyuki OJIMA, Shimato ONO, Yoshio OMATA, Sadanobu KAGAMIMORI, Takahiko KATOH, Masahiko KATO, Yukinori KUSAKA, Shinya KUNO, Kiyokage KUBO, Yoshiki KURODA, Yasuaki SAIJO, Eiji SHIBATA, Kuninori SHIWAKU, Narufumi SUGANUMA, Tomotaka SOBUE, Toshiro TAKEZAKI, Toru TAKEBAYASHI *, Atsuhiro TATEOKA, Masatoshi TANAKA, Tsuyoshi TANABE, Masakazu NAKAGUN, Susumu TSUKAMOTO, Hiroyuki DOI, Kunio DOBASHI, Chisato NAGATA, Hiroyuki NAKAMURA, Kunio NAKAYAMA, Norihiro NOGATA, Takashi HANATO, Daisuke FUKUDOME, Yoshihisa FUJINO *, Tanji HOSHI *, Satoshi HOSHIDE, Takahiro MAEDA, Toru MATSUDA, Muneo MINOSHIMA, Takashi MURAWAKA, Naoki YASUDA, Genichiro YATAKE, Hidekazu YAMADA, Shoji YOSHIDA, Misako YOSHINAGA, Masahito YONEOKA, Ken YONEDA  **Committee member in architecture**  　Akihiko IWASA, Atsushi IWAMAE *, Akihito OZAKI, Satoru KUNO, Minoru KUMANO, Shoichi KOJIMA, Yuzo SAKAMOTO, Yasuyuki SHIRAISHI, Hirotaka SUZUKI, Tsuyoshi SEIKE *, Naoki TAKAGI, Masaki TAJIMA, Yoshito TANAKA, Takayuki TAMAI, Mitsutaka TSUJI, Reiji TOMIKU, Hisaya NAGAI, Daisaku NISHINA, Hideyo NIMIYA, Kenichi HASEGAWA, Hirofumi HAYAMA *, Akira FUKUSHIMA, Yuji HORI, Kiyotada MAGATA, Takeo MATSUOKA, Teruaki MITAMURA, Shinji YOSHIDA |
| --- |

*: members of the Research Planning Committee for the Promotion of Smart Wellness Housing

B: Members of the Subcommittee for Analysis of the Smart Wellness Housing Survey

| **Chairperson**  　Toshiharu IKAGA *  **Vice-chairperson**  　Yoshihisa FUJINO *  **Organizer**  　Shintaro ANDO *, Tatsuhiko KUBO  **Committee member**  　Yuko OGUMA, Hiroshi KANEGAE, Shun KAWAKUBO, Yoshinobu SAITO, Masaru SUZUKI, Tsuyoshi SEIKE *  **Experts committee member**  　Maki ITO, Wataru UMISHIO, Hiroshi KOJIMA, Takayuki TAJIMA, Yukie NAKAJIMA  **Adviser**  　Kazuomi KARIO *, Toru TAKEBAYASHI *, Tanji HOSHI *, Takesumi YOSHIMURA * |
| --- |

*: members of the Research Planning Committee for the Promotion of Smart Wellness Housing

Table S2. Summary of S standards of the “Act on the Promotion of Dissemination of Long-Lasting Quality Housing” in Japan

| Route | Requirement | | | | |
| --- | --- | --- | --- | --- | --- |
|  | for outer skin † |  | for energy consumption ‡ |  | for openings § |
| 1 | Grade 4 of energy conservation |  |  |  |  |
| 2 | Grade 4 of thermal insulation |  |  |  |  |
| 3 | Grade 3 of energy conservation | + | Grade 4 of primary energy consumption  (not including solar energy equipment) |  |  |
| 4 | Grade 3 of thermal insulation | + | Grade 4 of primary energy consumption  (not including solar energy equipment) |  |  |
| 5 | Grade 3 of energy conservation | + | Grade 4 of primary energy consumption  (including solar energy equipment) | + | Specific heat insulation work on openings |
| 6 | Grade 3 of thermal insulation | + | Grade 4 of primary energy consumption  (including solar energy equipment) | + | Specific heat insulation work on openings |

† ‘Grade of energy conservation’ and ‘Grade of thermal insulation’ is shown in Table S3 and S4 in detail.

‡ ‘Grade 4 of primary energy consumption’ aims to reduce the primary energy consumption to the standard value of primary energy consumption or less.

§ ‘Specific heat insulation work on openings’ aims to reduce the average heat transmission coefficient for openings to the following values or less: 2.33 W/m^2^ in areas 1−3, 3.49 W/m^2^ in area 4, and 4.65 W/m^2^ in areas 5−7.

Table S3. Performance-based grades of energy conservation

| Grade | | Indicator † | | Area | | | | | | | |
| --- | --- | --- | --- | --- | --- | --- | --- | --- | --- | --- | --- |
|  |  |  |  | 1 | 2 | 3 | 4 | 5 | 6 | 7 | 8 |
| 4 | (1999 standards) | Q value | [W/m^2^K] | 1.6 | 1.6 | 1.9 | 2.4 | 2.7 | 2.7 | 2.7 | 3.7 |
|  |  | μ value | [-] | 0.08 | 0.08 | 0.08 | 0.07 | 0.07 | 0.07 | 0.07 | 0.06 |
|  |  | U_w_ value | [W/m^2^K] | 2.33 | 2.33 | 2.33 | 3.49 | 4.65 | 4.65 | 4.65 | 6.51 |
| 3 | (1992 standards) | Q value | [W/m^2^K] | 1.8 | 1.8 | 2.7 | 3.3 | 4.2 | 4.2 | 4.6 | 8.1 |
|  |  | μ value | [-] | － | － | － | 0.10 | 0.10 | 0.10 | 0.10 | 0.08 |
|  |  | U_w_ value | [W/m^2^K] | 2.33 | 2.33 | 3.49 | 4.65 | 6.51 | 6.51 | 6.51 | 6.51 |
| 2 | (1980 standards) | Q value | [W/m^2^K] | 2.8 | 2.8 | 4.0 | 4.7 | 5.2 | 5.2 | 8.3 | 8.3 |
|  |  | μ value | [-] | － | － | － | － | － | － | － | － |
|  |  | U_w_ value | [W/m^2^K] | － | － | － | － | － | － | － | － |

† Q value, heat loss coefficient (= Total amount of heat loss (including heat loss through ventilation) [W/K]/Total floor area [m^2^]); μ value, solar heat gain coefficient during summer season (= Solar heat gain [W/(W/m^2^)]/Total floor area [m^2^]); U_w_ value, heat transmission coefficient for windows (= Total amount of heat loss through window [W/K]/Total window area [m^2^])

Table S4. Performance-based grades of thermal insulation

| Grade | | Indicator † | | Area | | | | | | | |
| --- | --- | --- | --- | --- | --- | --- | --- | --- | --- | --- | --- |
|  |  |  |  | 1 | 2 | 3 | 4 | 5 | 6 | 7 | 8 |
| 4 | (2013 standards) | U_A_ value | [W/m^2^K] | 0.46 | 0.46 | 0.56 | 0.75 | 0.87 | 0.87 | 0.87 | － |
|  |  | η_AC_ value | [-] | － | － | － | － | 3.0 | 2.8 | 2.7 | 3.2 |
|  |  | U_w_ value ‡ | [W/m^2^K] | 2.33 | 2.33 | 2.33 | 3.49 | 4.65 | 4.65 | 4.65 | － |
| 3 | (1992 standards) | U_A_ value | [W/m^2^K] | 0.54 | 0.54 | 1.04 | 1.25 | 1.54 | 1.54 | 1.81 | － |
|  |  | η_AC_ value | [-] | － | － | － | － | 4.0 | 3.8 | 4.0 | 4.5 |
|  |  | U_w_ value ‡ | [W/m^2^K] | 2.33 | 2.33 | 3.49 | 4.65 | 6.51 | 6.51 | 6.51 | － |
| 2 | (1980 standards) | U_A_ value | [W/m^2^K] | 0.72 | 0.72 | 1.21 | 1.47 | 1.67 | 1.67 | 2.35 | － |
|  |  | η_AC_ value | [-] | － | － | － | － | － | － | － | － |
|  |  | U_w_ value ‡ | [W/m^2^K] | － | － | － | － | － | － | － | － |

† U_A_ value, average heat transmission coefficient for the outer skin (= Total amount of heat loss (not including heat loss through ventilation) [W/K]/Total outer skin area [m^2^]); η_AC_ value, solar heat gain coefficient during summer season (= Solar heat gain [W/(W/m^2^)]/Total outer skin area [m^2^] × 100); U_w_ value, heat transmission coefficient for windows (= Total amount of heat loss through window [W/K]/Total window area [m^2^])

‡ U_w_ value is the value when the ratio of opening area (= Total opening area [m^2^]/Total outer skin area [m^2^]) is within following range: 0.07−0.09 in areas 1−3, 0.08−0.11 in areas 4−8 for detached houses.

Table S5. Average living room temperature in the morning by resident characteristic

| Variable | *n* | Mean | Difference  from ref. | *p* value |
| --- | --- | --- | --- | --- |
| Age |  |  |  |  |
| <40 years (ref.) | 187 | 16.3 | ref. | – |
| 40–49 years | 302 | 16.2 | – 0.0 | 1.000 |
| 50–59 years | 531 | 15.0 | – 1.3 | 0.006 |
| 60–69 years | 685 | 14.7 | – 1.6 | <0.001 |
| 70–79 years | 348 | 14.9 | – 1.3 | 0.009 |
| ≥80 years | 132 | 14.4 | – 1.8 | 0.002 |
| Sex |  |  |  |  |
| Men (ref.) | 1,781 | 15.4 | ref. | – |
| Women | 404 | 14.2 | – 1.2 | <0.001 |
| BMI |  |  |  |  |
| Underweight (<18.5) (ref.) | 91 | 14.9 | ref. | – |
| Normal (18.5–25.0) | 1,513 | 15.1 | 0.3 | 0.816 |
| Obesity (>25.0) | 581 | 15.2 | 0.4 | 0.753 |
| Duration of residence in house |  |  |  |  |
| <10 years (ref.) | 336 | 16.5 | ref. | – |
| 10–19 years | 390 | 16.3 | – 0.2 | 0.996 |
| 20–29 years | 550 | 15.2 | – 1.3 | <0.001 |
| 30–39 years | 389 | 14.8 | – 1.7 | <0.001 |
| 40–49 years | 237 | 13.9 | – 2.6 | <0.001 |
| ≥50 years | 235 | 13.3 | – 3.1 | <0.001 |
| Household income |  |  |  |  |
| High (≥6 million JPY) (ref.) | 729 | 15.7 | ref. | – |
| Middle (2–6 million JPY) | 1,039 | 15.1 | – 0.6 | 0.020 |
| Low (<2 million JPY) | 259 | 14.2 | – 1.4 | <0.001 |
| Number of housemates |  |  |  |  |
| ≥2 (ref.) | 1,928 | 15.3 | ref. | – |
| 1 (single-person households) | 213 | 14.1 | – 1.2 | <0.001 |
| Amount of clothes |  |  |  |  |
| <1.00 clo† (ref.) | 805 | 16.2 | ref. | – |
| ≥1.00 clo† | 1,317 | 14.6 | – 1.6 | <0.001 |

Table S5. (continued)

| Variable | *n* | Mean | Difference  from ref. | *p* value |
| --- | --- | --- | --- | --- |
| Kotatsu‡ use |  |  |  |  |
| None (ref.) | 1,301 | 16.0 | ref. | – |
| Currently used | 860 | 13.9 | – 2.2 | <0.001 |
| Area |  |  |  |  |
| Area 6 (ref.) | 1,108 | 15.5 | ref. | – |
| Area 2 | 68 | 19.3 | 3.8 | <0.001 |
| Area 3 | 69 | 14.8 | – 0.7 | 0.803 |
| Area 4 | 249 | 13.7 | – 1.8 | <0.001 |
| Area 5 | 560 | 14.7 | – 0.8 | 0.004 |
| Area 7 | 130 | 15.0 | – 0.5 | 0.786 |

† clo is a unit that represents the thermal resistance of clothes. 1 clo=0.155(m^2^K)/W.

‡ traditional Japanese local heating device, consisting of a low table with an electric heater underneath and covered by a thick blanket.

Figure S1. Definition of heating degree days

† The effects of solar radiation or internal gains from lights, humans and appliances are not included in the definition of heating degree days.

Figure S2. Prefectures in Japan

Figure S3. Traditional Japanese heating device “kotatsu table”

Figure S4. Patterns of clothes in the questionnaire

† The amount of clothes was calculated as follows:

#1: 0.69 clo (= Panties: 0.03 +T-shirt: 0.08 +Long-sleeve flannel shirt: 0.34 +Straight trousers (thick): 0.24)

#2: 1.05 clo (= #1 +Long-sleeve sweater(thick): 0.36)

#3: 1.11 clo (= #1 +Single-breasted suit jacket(thick): 0.42)

#4: 0.67 clo (= Bra: 0.01 +Panties: 0.03 +Full slip: 0.16 +Long-sleeve shirtdress (thick): 0.47)

#5: 1.13 clo (= Bra: 0.01 +Panties: 0.03 +Full slip: 0.16 +Long-sleeve flannel shirt: 0.34 +Skirt (thick): 0.23 +Long-sleeve sweater (thick): 0.36)

#6: 1.19 clo (= Bra: 0.01 +Panties: 0.03 +Full slip: 0.16 +Long-sleeve flannel shirt: 0.34 +Skirt (thick): 0.23 +Single-breasted suit jacket (thick): 0.42)

#7: 0.68 clo (= Panties: 0.03 +T-shirt: 0.08 +Long-sleeve pajamas (thick): 0.57)

#8: 1.14 clo (= #1 +Long-sleeve long gown (thick): 0.46)

#9: 0.73 clo (= Panties: 0.03 +T-shirt: 0.08 +Long-sleeve sweatshirt: 0.34 +Sweatpants: 0.28)

#10: 1.19 clo (= #9 +Long-sleeve long gown (thick): 0.46)

#11: 0.69 clo (= Panties: 0.03 +T-shirt: 0.08 +Long-sleeve sweatshirt: 0.34 +Straight trousers (thick): 0.24)

#12: 0.89 clo（= Bra: 0.01 +Panties: 0.03 +Full slip: 0.16 +Long-sleeve long robe (thick): 0.69）

Figure S5. Maximum living room and changing room temperature at home

and Maximum bedroom temperature during sleep

† The bar chart indicates the number of houses and the line chart indicates cumulative frequency.

The result shows the 2-week average of daily maximum temperature for each house

when participants were at home (not during sleep) or were in bed.
