# Supplementary material for: Disparities of indoor temperature in winter: A cross‐sectional analysis of the Nationwide Smart Wellness Housing Survey in Japan
Source: Indoor Air. 2020 Jul 6;30(6):1317–28. doi: 10.1111/ina.12708 (PMC7689703; doi:10.1111/ina.12708)
Supplement: Supplementary file 2 — Supplementary Material [file INA-30-1317-s002.docx]

**Details of the multilevel model**

The multilevel linear regression models were as follows:

Day level (variables changing from day to day):

Temp_Lr_ _ij_ = *α*_0j_ + $\sum_{\text{ s=1}}^{\text{ p}} \text{α}_{\text{sj}}\text{× }\text{W}_{\text{ij}}$ + *r*_ij_

where Temp_Lr_ _ij_ is the observed living room temperature for household j on day i, *α*_0j_ is the random intercept, *α*_sj_ are regression coefficients for *W*_ij_ (day-level variable: outdoor temperature), and *r*_ij_ is the residual for household j on day i.

Household level (variables differing from household to household):

*α*_0j_ = *β*_00_ + $\sum_{\text{ }\text{t=1}}^{\text{ q}} \text{β}_{\text{0}\text{t}}\text{× }\text{X}_{\text{j}}$ + *u*_0j_

*α*_sj_ = *β*_s0_ (s=1, 2, 3, …)

where *β* is the fixed effect, *X*_j_ stands for each household-level variable (age, duration of residence in the same house, household income, single-person households, kotatsu use, and the amount of clothes and climate area), and *u*_0j_ is the random effect for household j.

The multilevel logistic regression models were as follows:

Day level (variables changing from day to day):

Logit(*p*_ij_) = *α*_0j_ + $\sum_{\text{ s=1}}^{\text{ p}} \text{α}_{\text{sj}}\text{× }\text{W}_{\text{ij}}$ + *r*_ij_

where *p*_ij_ is the probability of observed living room temperature falling to <18°C (the recommended minimum temperature by the WHO guidelines) for household j on day i. *α*_0j_ is the random intercept, *α*_sj_ are regression coefficients for *W*_ij_ (day-level variable: outdoor temperature), and *r*_ij_ is the residual for household j on day i.

Household level (variables differing from household to household):

*α*_0j_ = *β*_00_ + $\sum_{\text{ }\text{t=1}}^{\text{ q}} \text{β}_{\text{0}\text{t}}\text{× }\text{X}_{\text{j}}$ + *u*_0j_

*α*_sj_ = *β*_s0_ (s=1, 2, 3, …)

where *β* is the fixed effect, *X*_j_ stands for each household-level variable (age, duration of residence in the same house, household income, single-person households, kotatsu use, and the amount of clothes and climate area), and *u*_0j_ is the random effect for household j.
